# Cisgenic overexpression of cytosolic glutamine synthetase improves nitrogen utilization efficiency in barley and prevents grain protein decline under elevated CO_2_

**DOI:** 10.1111/pbi.13046

**Published:** 2018-12-27

**Authors:** Yajie Gao, Thomas C. de Bang, Jan K. Schjoerring

**Affiliations:** ^1^ Department of Plant and Environmental Sciences Faculty of Science Copenhagen University Frederiksberg Denmark

**Keywords:** Nitrogen use efficiency (NUE), glutamine synthetase (GS), cisgenesis, carbon dioxide (CO_2_), grain protein, barley

## Abstract

Cytosolic glutamine synthetase (GS1) plays a central role in nitrogen (N) metabolism. The importance of GS1 in N remobilization during reproductive growth has been reported in cereal species but attempts to improve N utilization efficiency (NUE) by overexpressing *GS1* have yielded inconsistent results. Here, we demonstrate that transformation of barley (*Hordeum vulgare* L.) plants using a cisgenic strategy to express an extra copy of native *HvGS1‐1* lead to increased *HvGS1.1* expression and GS1 enzyme activity. *GS1* overexpressing lines exhibited higher grain yields and NUE than wild‐type plants when grown under three different N supplies and two levels of atmospheric CO_2_. In contrast with the wild‐type, the grain protein concentration in the *GS1* overexpressing lines did not decline when plants were exposed to elevated (800–900 μL/L) atmospheric CO_2_. We conclude that an increase in GS1 activity obtained through cisgenic overexpression of *HvGS1‐1* can improve grain yield and NUE in barley. The extra capacity for N assimilation obtained by *GS1* overexpression may also provide a means to prevent declining grain protein levels under elevated atmospheric CO_2_.

## Introduction

Nitrogen (N) is essential for plant growth and a primary driver of crop yields. One of the main challenges for modern agriculture is to reduce the use of N fertilizers, while at the same time maintaining or improving grain yield and quality (Hawkesford, 2014; Hirel *et al*., 2011). Nitrogen is taken up by plants mainly as nitrate (NO_3_^−^) or ammonium (NH_4_^+^). Nitrate is first reduced to nitrite (NO_2_^−^) and subsequently to NH_4_^+^ by the actions of nitrate reductase and nitrite reductase. Glutamine synthetase (GS) thereafter catalyses the condensation of NH_4_^+^ and glutamate, thus constituting the first step in the biosynthesis of organic N compounds. In most plant species, GS occurs as a single isoform in the chloroplast (GS2) and up to five isoforms in the cytosol (GS1; Swarbreck *et al*., 2011). GS2 is mainly involved in assimilation of NH_4_^+^ originating from NO_3_^−^ reduction and photorespiration (Brestic *et al*., 2014; Igamberdiev *et al*., 2014; Pérez‐Delgado *et al*., 2015). Barley mutants lacking GS2 were unable to grow under normal atmospheric conditions as they were not able to re‐assimilate photorespiratory ammonium and accumulated relatively high levels of ammonium in the leaves (Blackwell *et al*., 1987; Wallsgrove *et al*., 1987). GS1 plays a role in primary NH_4_^+^ assimilation in the roots (Funayama *et al*., 2017; Guan *et al*., 2016; Konishi *et al*., 2018) and is, in addition, involved in the assimilation and recycling of NH_4_^+^ released from protein degradation during senescence (Avila‐Ospina *et al*., 2014; Bernard and Habash, 2009; Krapp, 2015).

Individual GS1 isoforms may exert different functions reflecting their kinetic properties and expression patterns. The barley (*Hordeum vulgare* L.) genome encodes three GS1 isozymes of which *HvGS1‐1* transcripts are present mainly in the vascular tissue, *HvGS1‐2* in the root and *HvGS1‐3* almost exclusively in grains (Goodall *et al*., 2013). GS1 isozymes in wheat (*Triticum aestivum* L.) are encoded by three sub‐families, *viz. TaGS1 (a, b and c)*,* TaGSe (1 and 2)* and *TaGSr (1 and 2)* (Bernard *et al*., 2008). *TaGS1* is the orthologue of *HvGS1‐1* and is up‐regulated during flowering and grain development (Bernard *et al*., 2008). The corresponding orthologue in maize (*Zea mays* L.), *ZmGln1‐4*, is localized in the bundle sheath cells and up‐regulated during senescence (Martin *et al*., 2006). A rice (*Oryza sativa* L.) mutant lacking the *HvGS1‐1* orthologue *OsGS1‐1* showed severe reduction in grain yield (Tabuchi *et al*., 2005). Studies of quantitative trait loci have indicated that the barley *HvGS1‐1* gene is associated with grain protein concentration (Jukanti and Fischer, 2008), while the maize orthologues *ZmGln1‐3* and *ZmGln1‐4* are associated with kernel weight and grain yield, respectively (Gallais and Hirel, 2004). A positive correlation between grain protein concentration, *GS* (*GS1* and *GS2*) gene expression and GS activity in durum wheat (*Triticum durum* L.) were reported by Nigro *et al*. (2016) and Zhang *et al*. (2017).

Attempts to improve the growth and N utilization efficiency (NUE) of plants by overexpressing *GS1* using constitutive promoters have yielded inconsistent results (Lu *et al*., 2018; Seger *et al*., 2015; Thomsen *et al*., 2014; Urriola and Rathore, 2015). This unpredictability may be a consequence of undesirable pleiotropic effects coming from the use of constitutive promoters or post‐translational regulation affecting enzyme activity. A further level of regulation may result from the fact that N assimilation is highly dependent on carbon (C) metabolism for the provision of energy and substrates (Osanai *et al*., 2017; Rubio‐Asensio and Bloom, 2017; Takabayashi *et al*., 2016). Despite this interconnection, grain protein concentrations have in several cases been shown to decline under elevated CO_2_ (Ainsworth and Long, 2005; Fernando *et al*., 2017; Högy and Fangmeier, 2008; Ingvordsen *et al*., 2016; Wroblewitz *et al*., 2014). Application of additional N fertilizer to wheat plants growing in a free air CO_2_ enrichment facility was not able to counteract the decline in grain protein (Tausz *et al*., 2017), thus emphasizing the associated biochemical limitations. Overexpression of *GS1* might be a way to bypass this limitation.

As an alternative to transgenic approaches, the cisgenesis concept was introduced by Schouten *et al*. (2006) and Jacobsen and Schouten (2007). According to this concept, cisgenic plants can be genetically modified only with genetic material from the sexually compatible gene pool. Also, the cisgene has to be an identical copy of the endogenous gene in the normal‐sense orientation. Cisgenesis is considered to be one of the key breeding technologies that may ensure future progress in crop improvement (Bradshaw, 2017) and was recently used to achieve light blight resistance in potato by transforming a susceptible cultivar with resistance genes from wild potato species (Haverkort *et al*., 2016). The effects of cisgenic overexpression of genes involved in nitrogen metabolism have so far not been investigated.

The objective of the present work was to investigate how cisgenic overexpression of the native cytosolic glutamine synthetase gene *HvGS1‐1*, including its promoter, full‐length coding region and terminator, would affect grain yield and nitrogen use efficiency in barley. We hypothesized that this strategy would lead to consistent positive effects on plant performance through better alignment between the spatiotemporal expression pattern of *HvGS1‐1* and plant developmental requirements as compared to constitutive overexpression. We also hypothesized that cisgenic overexpression of *GS1* would counteract a decline in grain protein levels under elevated atmospheric CO_2_ as extra capacity for nitrogen assimilation would be provided.

## Results

### Genotyping and initial characterization of HvGS1‐1 cisgenic lines

The cisgenic locus number was determined by analysis of the segregation pattern of the inserted gene. In T1, six of 12 lines showed segregation for only one inserted locus and these were further genotyped to identify homozygous lines for single cisgenic loci in T2 (Table S1). The lines 2.2, 4.5, 11.1 and 11.3 were propagated to T3 and characterized in a greenhouse experiment (Figure S1). Higher grain yields relative to the wild‐type were observed for lines 2.2, 4.5 and 11.1 (*P* = 0.06, 0.09 and 0.18, respectively).

Two of the cisgenic lines (2.2 and 4.5), homozygous for a single cisgenic locus, were used in the further work due to their significantly (*P* < 0.05) better nitrogen use efficiency (NUE) expressed as the production of grain dry matter per unit of shoot N (Figure S1).

### *HvGS1‐1 expression and* HvGS1 *activity in HvGS1‐1 cisgenic lines*

During early vegetative growth, 28 days after germination (DAG), a 2–3 fold increase in *GS1‐1* expression relative to the wild‐type was observed in the two youngest fully developed leaves of the cisgenic lines relative to the wild‐type (Figure S2). Ion exchange chromatography was used to separate the cytosolic GS (GS1) from the chloroplastic GS (GS2) isoform. Two peaks of GS activity were detected and Western‐blot analysis used to confirm that the first peak consisted of GS1 and the second peak of GS2 (Figure S3). The GS1 activity in the two youngest fully developed leaves 28 DAG was about twofold higher than in the wild‐type thus matching the increase in *GS1‐1* expression (Figure S2).

**Figure 1:**
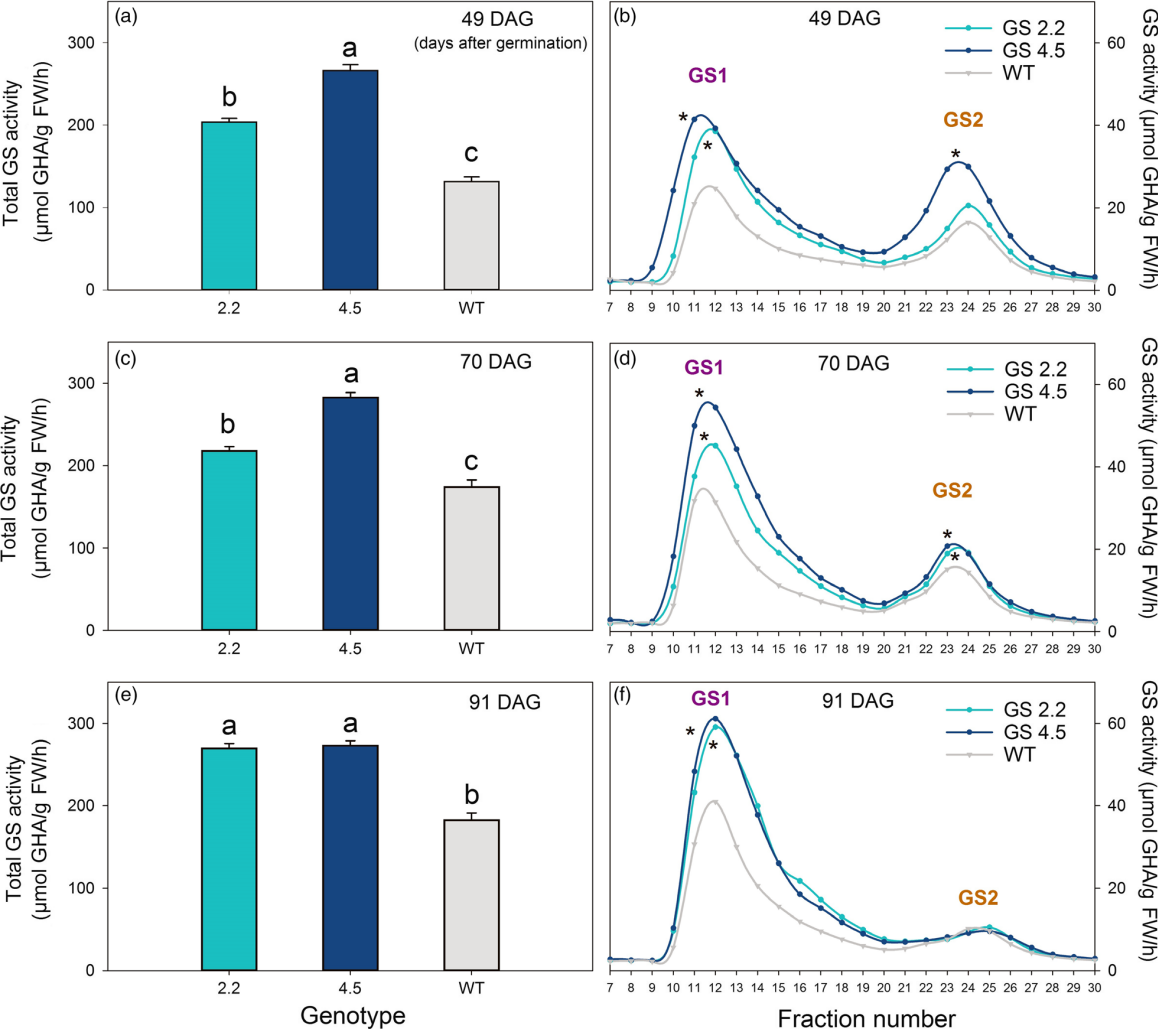
GS activity of two *HvGS1‐1* cisgenic lines (T3, 2.2 and 4.5) and wild‐type grown in soil with high N supply (0.6 g N/L soil). (a) Total GS activity in the two youngest fully developed leaves of plants at 49 days after germination (DAG; before ear emergence). (c) Total GS activity in the two upper leaves (flag leaf and the second leaf) from the main stem of plants at 70 DAG (after ear emergence) and (e) at 91 DAG (grain filling). Data represents mean values ± SE, *n* = 6. Different letters indicate significant difference (*P* < 0.05, Fischer LSD) between cisgenic lines and wild‐type. (b) Separate activities of cytosolic GS (GS1) and chloroplastic GS (GS2) in the two youngest fully developed leaves of plants at 49 DAG. (d) Separate activities of GS1 and GS2 in the two upper leaves from mean shoots of plants at 70 DAG and (f) at 91 DAG. The first peak corresponds to GS1 and the second peak to GS2 as confirmed by western‐blot analysis (Figure S3). GS1 and GS2 were separated on a Mono Q 5/50 GL anion column using Fast protein liquid chromatography (FPLC). Their activities in the different elution fractions were measured by the transferase assay and the produced γ‐GHA (γ‐glutamyl hydroxamate) quantified spectrophotometrically at 540 nm using synthetic GHA to prepare calibration standards. Symbols * close to the peak indicates significant difference of GS1 or GS2 activity between the cisgenic lines and wild‐type.

**Figure 2:**
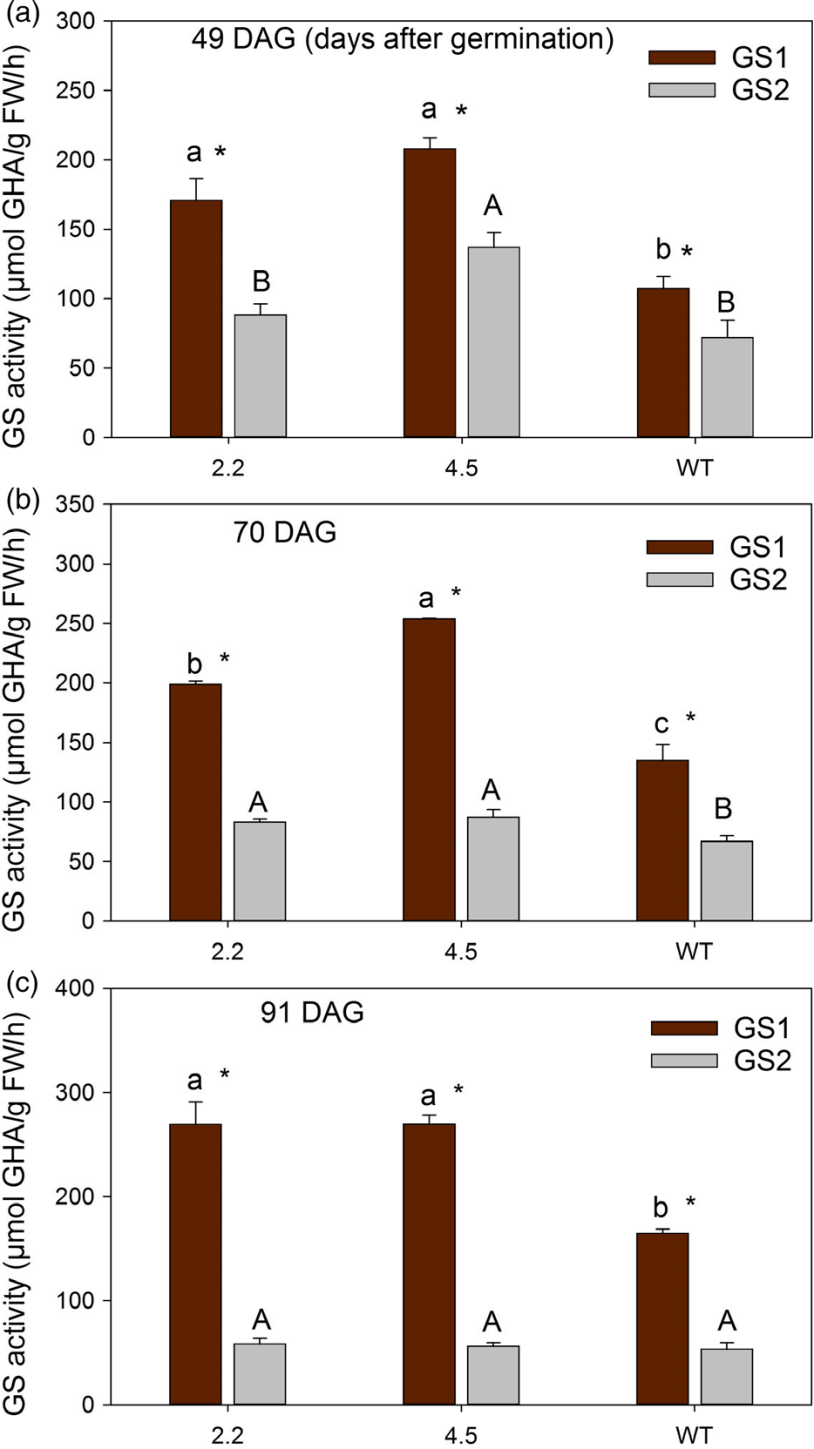
Separate activities of GS1 and GS2 in two *HvGS1‐1* cisgenic lines (T3, 2.2 and 4.5) and wild‐type grown in soil with high N supply (0.6 g N/L soil). (a) GS1 and GS2 activity in the two youngest fully developed leaves of plants at 49 days after germination (DAG; before ear emergence). (b) GS1 and GS2 activity in the two upper leaves (flag leaf and the second leaf) from the main stem of plants at 70 DAG (after ear emergence) and (c) at 91 DAG (grain filling). Data represents mean values ± SE (*n* = 4). Different letters indicate significant difference (*P* < 0.05, Fischer LSD) between cisgenic lines and wild‐type (lower letters and capital letters for GS1 and GS2 activity, respectively). Symbols * indicate significant difference between GS1 and GS2 activity of each genotype.

Also in later growth stages, i.e. just before ear emergence at 49 DAG, after ear emergence at 70 DAG and during grain filling at 91 DAG, the activity of GS1 was quantified to be 50%–100% higher in the cisgenic lines compared to wild‐type (Figures 1 and 2). A higher activity of GS2 was also measured in the two upper leaves at 49 and 70 DAG (Figure 1b, d; Figure 2a, b), but not at 91 DAG (Figure 1f). The total GS activity in the two upper leaves was throughout the period (49‐91 DAG) consistently higher in the cisgenic lines compared to the wild‐type (Figure 1).

### Effect of different N levels on yield structure and N economy of the HvGS1‐1 cisgenic lines

**Figure 3:**
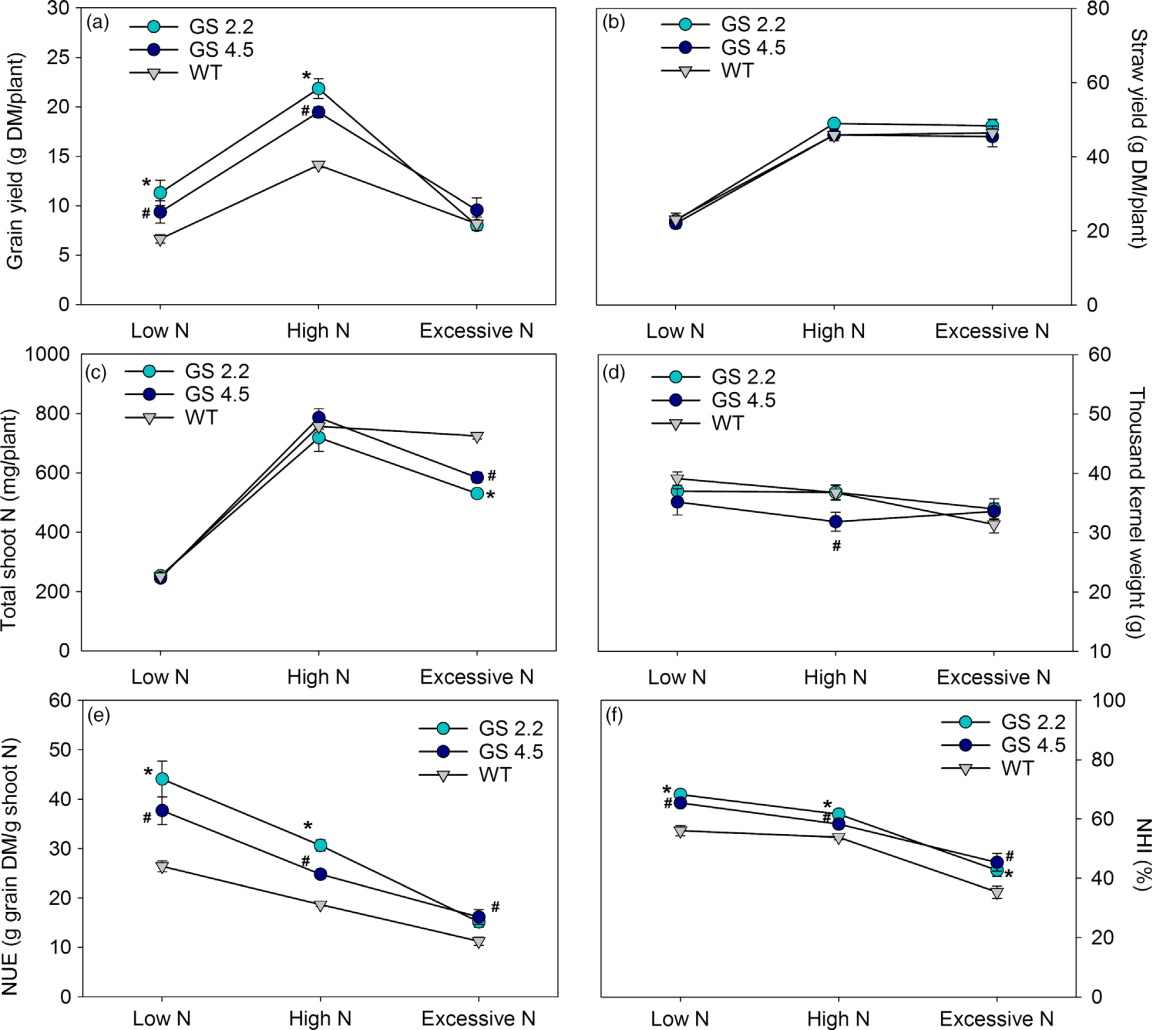
Grain yield and N use parameters at maturity of two *HvGS1‐1* cisgenic lines (T3, 2.2 and 4.5) and wild‐type under low, high and excessive N supply. (a) Grain yield, (b) straw yield (shoot biomass without grain), (c) total shoot N, (d) thousand kernel weight, (e) nitrogen utilization efficiency (NUE) and (f) nitrogen harvest index (NHI) of plants grown with low (0.2 g N/L soil), high (0.6 g N/L soil) and excessive (1.0 g N/L soil) N supply. Data are presented as means ± SE (*n* = 6). Significant differences (*P *< 0.05, Fischer LSD) between *HvGS1‐1* cisgenic lines and wild‐type inside each N treatment are indicated by # or * for line 4.5 and 2.2, respectively.

To further investigate the interactions between N supply, yield structure and N utilization, the two cisgenic lines and the wild‐type were grown to maturity in soil under greenhouse conditions at three different levels of N. No visual developmental differences appeared. At maturity, the cisgenic lines showed 41%–71% and 26%–42% higher grain yield compared to the wild‐type under low (0.2 g N/L soil) and high N (0.6 g N/L soil) supply, respectively (Figure 3a). When supplied with excessive N (1.0 g N/L soil), the grain yield in all cases decreased relative to the low N level, and the cisgenic lines no longer performed better than the wild‐type (Figure 3a). Excessive N supply was thus relatively more detrimental to the cisgenic lines than to the wild‐type. There were no differences in straw yields (Figure 3b).

The higher grain yields of the cisgenic lines at low (0.2 g N) and high (0.6 g N) N supply were achieved despite that their total shoot N content was similar to the wild‐type (Figure 3c). The cisgenic lines thus showed higher NUE than the wild‐type, corresponding to 35%–67% and 33%–44% more grain dry matter produced per unit of shoot N in line 2.2 and line 4.5, respectively (Figure 3e). Both of the cisgenic lines also had significantly higher N harvest index (NHI) compared to wild‐type (Figure 3f), while differences in thousand kernel weight were less pronounced (Figure 3d).

**Figure 4:**
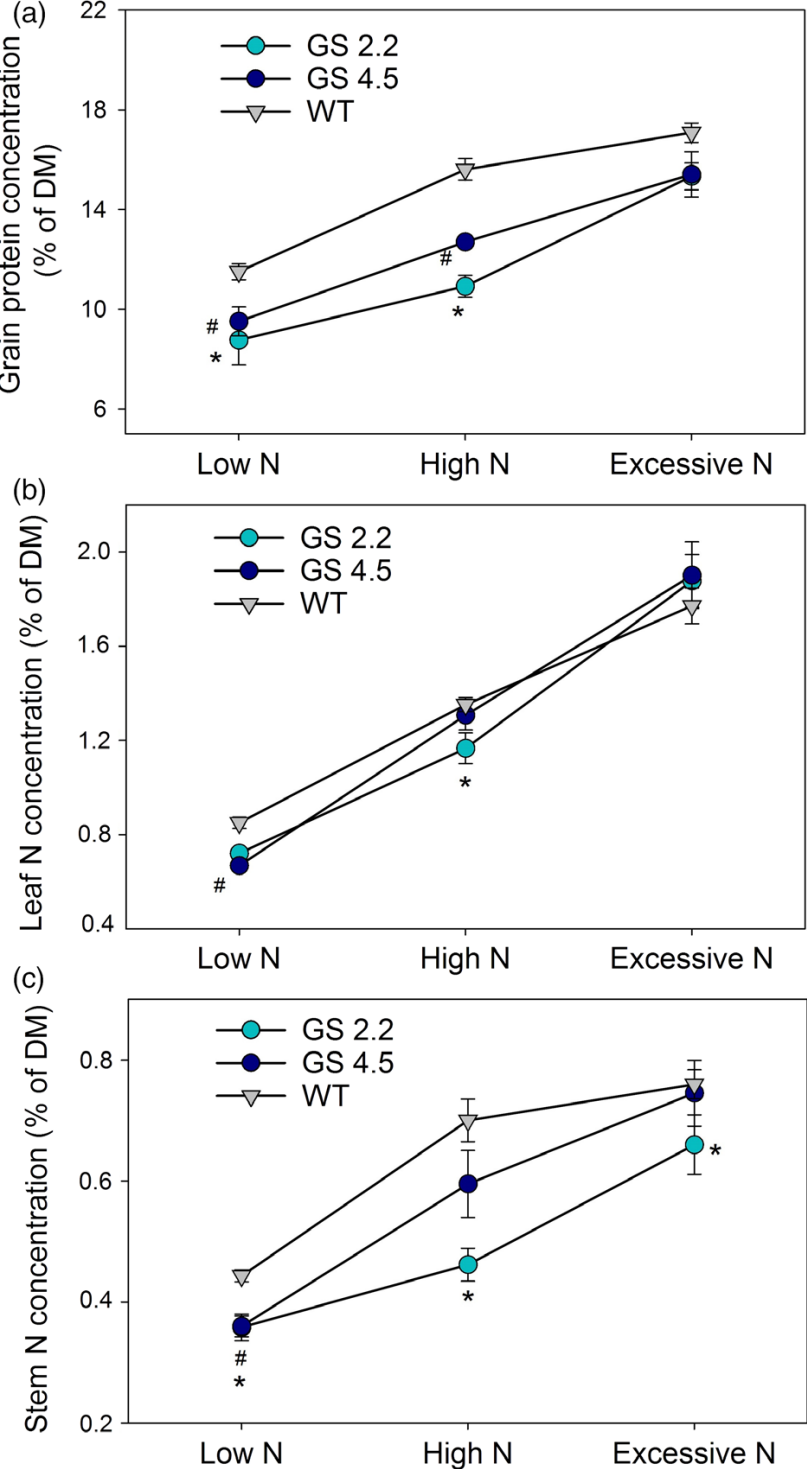
Grain protein concentration and N concentration in leaf and stem at maturity of two *HvGS1‐1* cisgenic lines (T3, 2.2 and 4.5) and wild‐type grown under low (0.2 g N/L soil), high (0.6 g N/L soil) and excessive (1.0 g N/L soil) N supply. (a) Grain protein concentration, (b) stem N concentration and (c) leaf N concentration. Data are presented as means ± SE (*n* = 6). Significant differences (*P* < 0.05, Fischer LSD) between cisgenic lines and the wild‐type inside each N treatment are indicated by # or * for line 4.5 and 2.2, respectively.

In accordance with the above differences in grain yield and NUE, the protein concentrations in grain dry matter of the two cisgenic lines grown at low or high N supplies were lower compared to wild‐type (Figure 4a). Also the corresponding N concentrations in leaves (Figure 4b) and stems (Figure 4c) were in most cases lower for the cisgenic lines.

### Effect of elevated atmospheric CO_2_ on yield structure and N economy of HvGS1‐1 cisgenic lines

**Figure 5:**
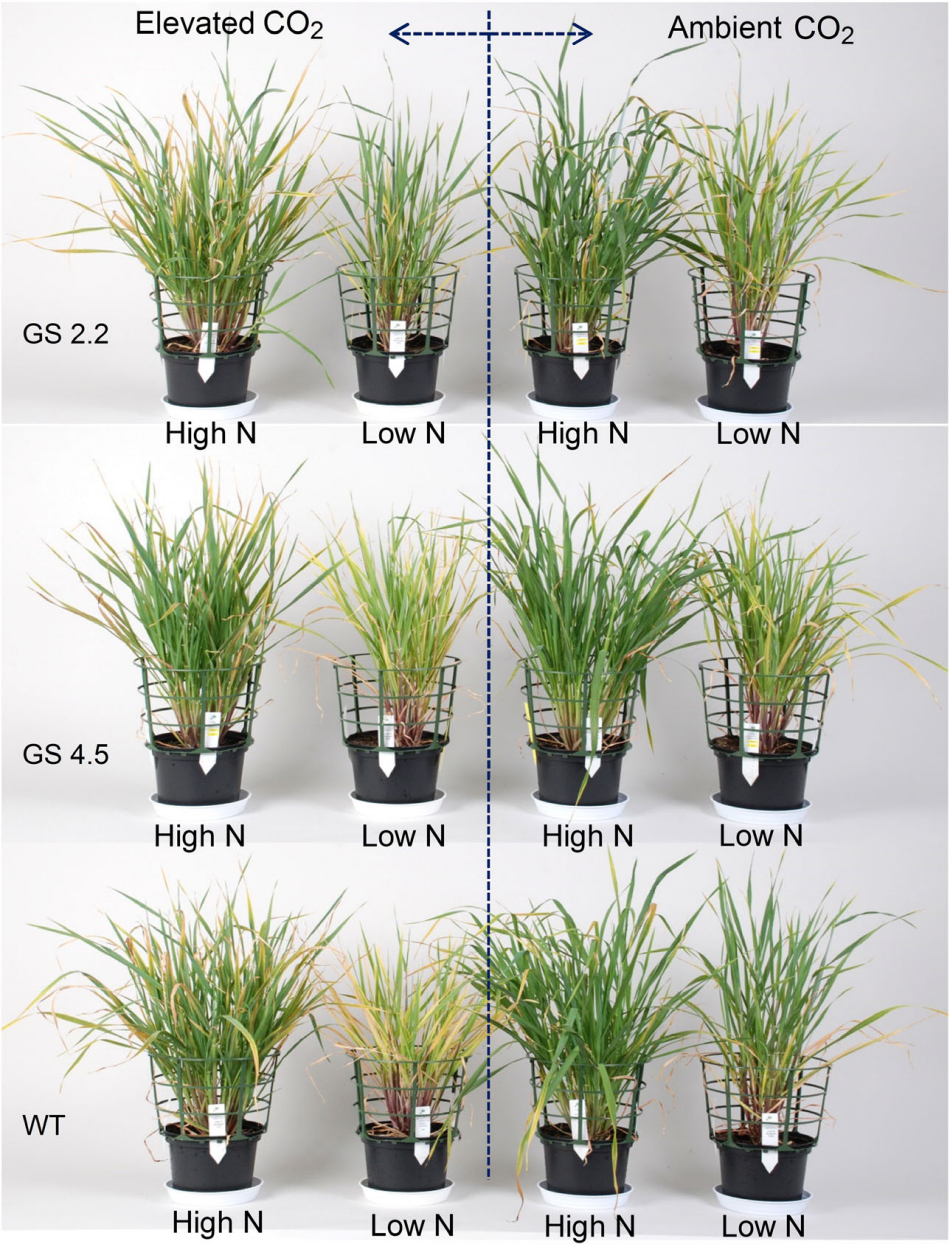
*HvGS1‐1* cisgenic lines (T3, 2.2 and 4.5) and wild‐type barley plants at 60 days after germination (DAG) growing at ambient (400 μL/L) or elevated (900 μL/L) atmospheric CO_2_ with low (0.2 g N/L soil) and high (0.6 g N/L soil) N supply.

**Figure 6:**
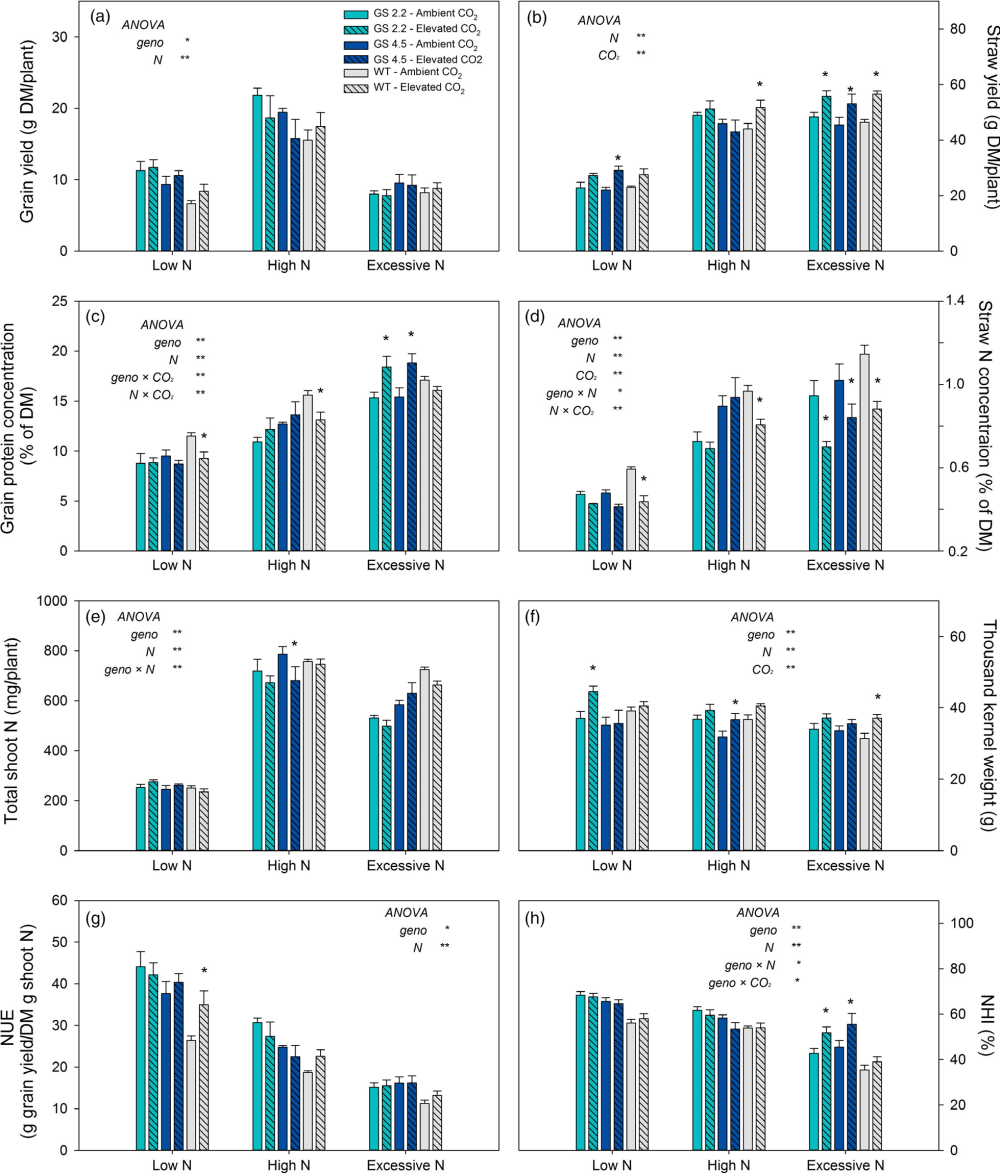
Grain yield and N use parameters at maturity of two *HvGS1‐1* cisgenic lines (T3, 2.2 and 4.5) and wild‐type growing under ambient (400 μL/L, open bars) or elevated (900 μL/L, hatched bars) atmospheric CO_2_. (a) Grain yield, (b) straw yield (shoot biomass without grain), (c) grain protein concentration, (d) straw N concentration, (e) total shoot N, (f) thousand kernel weight, (g) nitrogen utilization efficiency (NUE) and (h) nitrogen harvest index (NHI) of plants grown with low (0.2 g N/L soil), high (0.6 g N/L soil) and excessive (1.0 g N/L soil) N supply. Data are presented as means ± SE (*n* = 6). Asterisks (*) above bars indicate significant difference (*P* < 0.05, Fischer LSD) between ambient and elevated CO_2_ treatment inside each genotype. Levels of significance treatment effects (genotype, N and CO_2_ level) and their interactions analysed by ANOVA are shown as * (*P* < 0.05) and ** (*P* < 0.01), non‐significant effects are not indicated.

The *HvGS1‐1* cisgenic lines and the wild‐type plants were grown to maturity under two levels of atmospheric CO_2_ (ambient versus 900 μL/L) combined with three N levels. The purpose was to investigate if increased GS1 activity would benefit from the extra C skeletons provided by elevated atmospheric CO_2_ and thereby maintain grain protein levels under CO_2_ enrichment. Already during vegetative growth, stronger visual N deficiency symptoms appeared under elevated than under ambient CO_2_ and wild‐type plants seemed to be slightly more negatively affected by elevated CO_2_ than the cisgenic lines (Figure 5). At maturity, elevated CO_2_ resulted in higher straw yields rather than grain yields in both the cisgenic lines and wild‐type (Figure 6a, b). Also the thousand kernel weight was higher (Figure 6f), while the straw N concentration was lower (Figure 6d). The two cisgenic lines maintained the grain protein concentration under elevated CO_2_ in contrast to the wild‐type which had 20% and 15% lower grain protein concentration under low and high N level, respectively (Figure 6c). There was, thus, a significant interaction between the different genotypes and the CO_2_ level with respect to grain protein concentration. When supplied with excessive N, the cisgenic lines further benefitted from the elevated CO_2_, showing a 20% higher grain protein concentration than under ambient CO_2_ (Figure 6c). In general, elevated CO_2_ had no significant effect on total shoot N, NUE and NHI (Figure 6e, g, h). An exception was that the NHI of the cisgenic lines increased in response to elevated CO_2_ supplied with excessive N, which was due to the increased grain protein concentration (Figure 6h, c).

**Figure 7:**
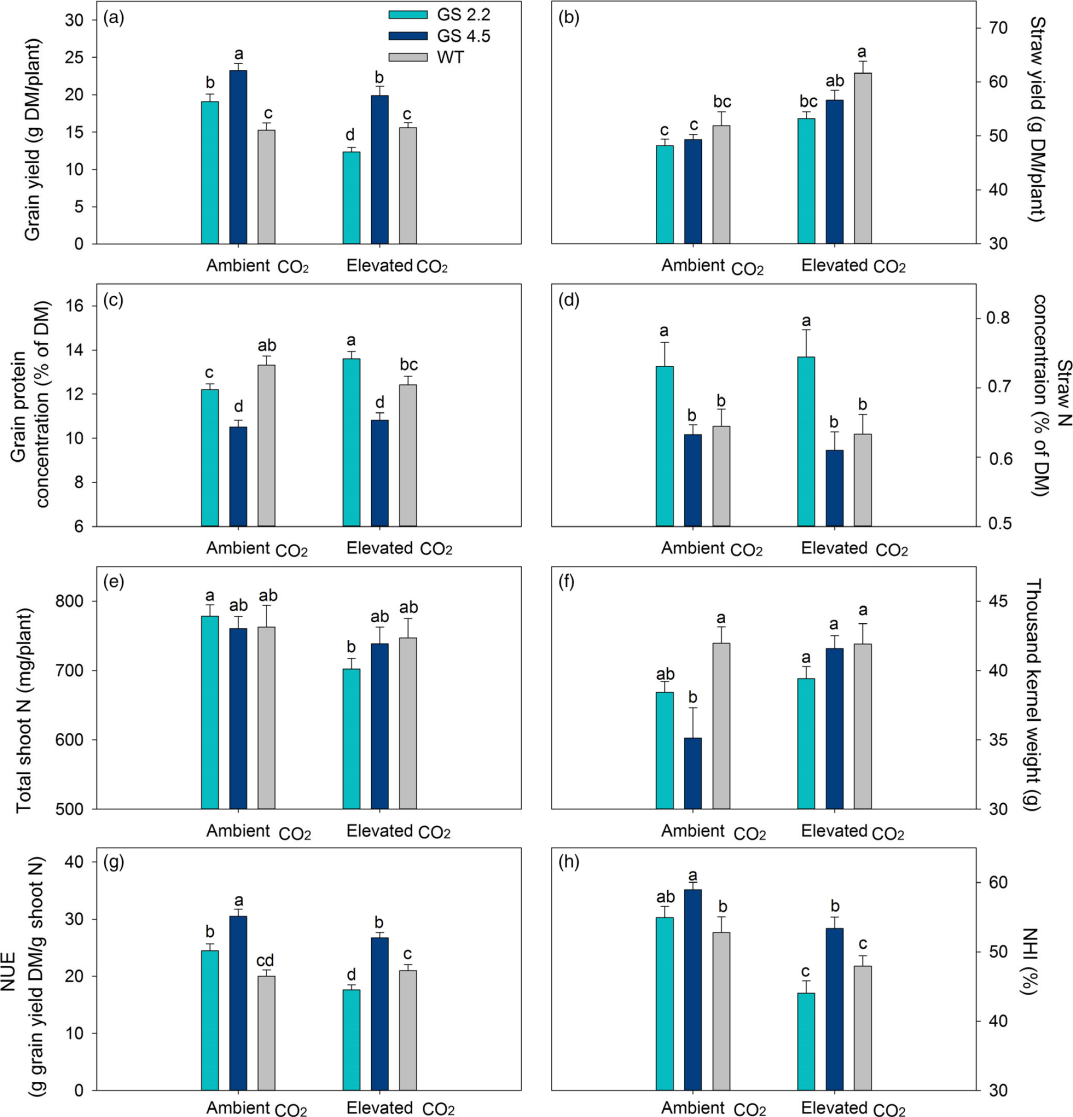
Grain yield and N use parameters at maturity of two *HvGS1‐1* cisgenic lines (T3, 2.2 and 4.5) and wild‐type growing under ambient (400 μL/L) or elevated (800 μL/L) atmospheric CO_2_. (a) Grain yield, (b) straw yield (shoot biomass without grain) (c) grain protein concentration, (d) straw N concentration, (e) total shoot N, (f) thousand kernel weight, (g) nitrogen utilization efficiency (NUE) and (h) nitrogen harvest index (NHI) of plants grown with medium (0.45 g N/L soil) N supply. Data are presented as means ± SE (*n* = 9). Different letters above bars indicate significant difference (*P* < 0.05, Fischer LSD) between genotypes under different CO_2_ treatment.

In order to provide an independent replication of the previous experiment, the *HvGS1‐1* cisgenic lines and the wild‐type plants were cultivated under two levels of atmospheric CO_2_ (ambient versus 800 μL/L) with a medium N level (0.45 g N/L soil). The results obtained under ambient atmospheric CO_2_ in the new experiment again showed significantly higher grain yields and NUE of the cisgenic lines compared to the wild‐type (Figure 7a, g). Lower grain protein concentrations of the cisgenic lines relative to the wild‐type under ambient CO_2_ was also confirmed (Figure 7c), while straw yield and total shoot N content (grain plus straw) did not differ between the cisgenic lines and the wild‐type (Figure 7b, e).

Both the two cisgenic lines and the wild‐type plants responded to elevated CO_2_ by increasing the straw yield rather than the grain yield (Figure 7a, b). Actually, the grain yield of both cisgenic lines declined relative to ambient CO_2_. This was due to a lower grain number per spike (data not shown), while the thousand kernel weight increased in response to elevated CO_2_ (Figure 7f). In spite of the larger grain size, the grain protein concentration in the cisgenic lines did not decline under elevated CO_2_, whereas that in the wild‐type showed a 7% decrease (*P* = 0.12). The grain protein concentration of line 2.2 in fact increased 12% in response to elevated CO_2_ and became significantly higher than that in the wild‐type (Figure 7c). The other cisgenic line (line 4.5) still had a lower grain protein concentration than the wild‐type, but this line performed significantly better than the wild‐type in terms of grain yield, NUE and NHI under elevated CO_2_ (Figure 7a, g, h). The straw N concentration was not affected by elevated CO_2_ in neither the cisgenic lines nor the wild‐type (Figure 7d).

## Discussion

In the present work, we inserted one extra copy of the native *HvGS1‐1* cisgene into barley, thus following the cisgenesis concept introduced by Schouten *et al*. (2006) and Jacobsen and Schouten (2007). However, we did not analyse the transformed barley lines for the presence of selection marker genes and vector‐backbone sequences. Thus, the lines obtained were not fully developed cisgenic lines but, nevertheless, the underlying biological mechanisms affecting the outcome of the cisgenic strategy are still reflected.

### Gene expression and enzyme activity in HvGS1‐1 cisgenic lines

Transgenic approaches to overexpress *GS1* driven by strong constitutive or tissue‐specific promoters have yielded inconsistent results (Lu *et al*., 2018; Seger *et al*., 2015; Thomsen *et al*., 2014; Urriola and Rathore, 2015). There may be several reasons for this, including negative pleiotropic effects, post‐translational regulation and C‐N imbalances (Thomsen *et al*., 2014). In the present work, we used a cisgenic approach in which barley plants were transformed with the genomic clone of the *HvGS1‐1* gene embracing the promoter and the full‐length transcript of the *HvGS1‐1* gene. This was done in an attempt to increase gene expression and enzyme activity within the native background of regulation across different tissues and experimental conditions. This strategy appeared successful since both the *GS1‐1* expression and GS1 activity in the cisgenic lines increased about twofold (Figures 1 and 2; Figure S2). Thus, there was seemingly no significant post‐translational regulation preventing the increase in gene expression from resulting in an increased enzyme activity in the cisgenic lines.

A concomitant increase in GS2 and GS1 activities were observed in the *HvGS1‐1* overexpressing lines, mainly in the two upper leaves before and during ear emergence (Figure 1b, d), but not at the later grain filling stage (Figure 1f). A similar positive interaction between GS1 and GS2 activities in young leaves was observed in transgenic barley plants overexpressing the *HvGS1‐1* gene driven by a constitutive promoter (Gao *et al*., 2018). The increase in GS2 activity may reflect different levels of regulation affecting the assembly of the GS1 and GS2 holoenzymes (Wang *et al*., 2015) or the association of GS2 or GS1 polypeptides with other proteins (Seabra and Carvalho, 2015; Seabra *et al*., 2013). Overall, the interaction between GS1 and GS2 may serve to balance N and C metabolism (Bao *et al*., 2014; Németh *et al*., 2018).

Throughout the period from ear emergence until grain filling (49‐91 DAG), the activity of GS1 exceeded that of GS2 (Figure 2). The differences in GS1 and GS2 activity actually became more pronounced during the period due to increasing GS1 and declining GS2 activities (Figures 1 and 2). These changes reflect the degradation of chloroplastic proteins, including GS2, during senescence along with an up‐regulation of cytosolic GS1 for N re‐mobilization (Goodall *et al*., 2013; Kichey *et al*., 2005; Martinez *et al*., 2008).

### Yield structure and N use efficiency of HvGS1‐1 cisgenic lines

The present study is the first attempt to express a native *HvGS1‐1* gene in a crop species. Consistent improvements of grain yields, NHIs and NUEs were obtained (Figure 3). It is well‐established that cytosolic GS plays an important role in the remobilization of N from senescing leaves (Habash *et al*., 2007; Hirel *et al*., 2007; Obara *et al*., 2001, 2004). The higher activities of GS1 and total GS in the senescing leaves obtained by *HvGS1‐1* overexpression (Figure 1), thus signify an improved ability to remobilize N from senescing leaves, resulting in lower residual stem N concentrations (Figure 4) and higher NHIs (Figure 3) in the cisgenic lines. Along with having an effect on the absolute quantity of N remobilized, GS1 may also play a role for establishment of the actual yield capacity by maintaining sufficient N flows during critical growth stages (Guan *et al*., 2014; Thomsen *et al*., 2014). In maize, *ZmGln1;3* and *ZmGln1;4* played specific roles in the development of the cob with respect to kernel number and kernel size, respectively (Cañas *et al*., 2010; Martin *et al*., 2006). Cytokinin biosynthesis was recently shown to be positively correlated with GS activity (Ohashi *et al*., 2017) and cytokinins may constitute part of the signalling chain leading to growth stimulation by *GS1* overexpression.

Improved N economy and growth of the cisgenic lines were observed at low and high N levels, but not at excessive N supply (Figure 3). The N concentrations measured in the grain and straw ranged in the same intervals as normally encountered under field conditions when the N supply ranges from sub‐optimal to high levels. The toxic effects of excessive N supply actually turned out to be more detrimental for the cisgenic lines than for the wild‐type. This emphasizes that the outcome of *GS1* overexpression depends on a delicate balance between N and C and that this dependency may be part of the reason for the large number of inconsistent results obtained in previous *GS1* overexpression studies (Bao *et al*., 2014; Seger *et al*., 2015; Thomsen *et al*., 2014; Urriola and Rathore, 2015). The effect of constitutive overexpression of *HvGS1‐1* on grain yield and protein concentration in two transgenic barley lines was investigated by Gao *et al*. (2018). One of the lines had a dramatically increased GS1 activity in both young and senescing leaves as well as in the developing ear, but this resulted in a significant grain yield penalty. In contrast, the other line with a more modest increase in GS1 activity (15%–19%) showed steady improvements in grain yield (Gao *et al*., 2018). The results obtained in the present work indicate that employment of a cisgenic strategy in which *GS1* overexpression is subject to the native regulation mechanisms may lead to a more consistent positive result, at least as long as a reasonable N supply is provided.

### Effects of elevated atmospheric CO_2_ on the HvGS1‐1 cisgenic lines

The positive effect on grain yield obtained by cisgenic *GS1‐1* overexpression under ambient atmospheric CO_2_ could not be further stimulated by elevated atmospheric CO_2_ (Figures 6a, 7a). On the contrary, the grain yield of the two cisgenic lines declined in response to elevated CO_2_ (Figure 7a), while the straw yields increased (Figures 6b, 7b). The lower grain yields were due to a reduction in the number of grains per spike (data not shown). These changes indicated that photosynthates were not efficiently transported from leaves to spikes, reflecting the importance of the sink strength of the spike for the responsiveness of grain yields to elevated CO_2_ (Aranjuelo *et al*., 2011, 2013; Uddling *et al*., 2008). Nevertheless, the grain protein concentration in both of the cisgenic lines did not decline under elevated CO_2_ (Figures 6c, 7c) and was even increased in response to elevated CO_2_ when excessive N was applied (Figure 6c). This was the case despite the fact that the thousand kernel weight of both cisgenic lines increased under elevated CO_2_ (Figures 6f, 7f), which would be expected to decrease the protein concentration due to dilution with carbohydrates (Gifford *et al*., 2000; Pleijel and Uddling, 2012).

Exposure of plants to elevated CO_2_ has in a large number of cases been shown to result in a considerable reduction in the N concentration in different tissues, including the grain (Ainsworth and Long, 2005; Fernando *et al*., 2017; Högy and Fangmeier, 2008; Ingvordsen *et al*., 2016; Wroblewitz *et al*., 2014). The mechanisms underlying this decrease are not fully clear. Elevated CO_2_ has been shown to impede NO_3_^−^ reduction (Bloom, 2015; Bloom *et al*., 2014), thus negatively affecting the generation of NH_4_^+^ for subsequent incorporation in amino acids. In support of this biochemical bottleneck it was observed that NO_3_^−^ constituted a higher proportion of total N in plants exposed to elevated CO_2_ (Bahrami *et al*., 2017) and that additional N fertilizer was not able to counteract the decline in grain and tissue N concentrations in wheat (Tausz *et al*., 2017). A possible way to sustain tissue N concentrations and growth responses under elevated CO_2_ might be to supply plants with NH_4_^+^ instead of NO_3_^−^ (Rubio‐Asensio and Bloom, 2017). However, wheat plants taking up NH_4_^+^ instead of NO_3_^−^ in a free air CO_2_ enrichment facility did not respond with better growth and N acquisition (Dier *et al*., 2017). In spite of increasing thousand kernel weight, the grain protein concentration in the cisgenic lines increased under elevated CO_2_ (Figures 6c, 7c), suggesting that cisgenic *GS1* overexpression partially was able to circumvent biochemical bottlenecks in N assimilation. This may be the case even though down‐regulation of *GS1* expression under CO_2_ enrichment has been reported for different *TaGS1* isogenes in wheat (Buchner *et al*., 2015; Vicente *et al*., 2016, 2017), possibly reflecting a decrease in NH_4_^+^ (El Omari *et al*., 2010; Funayama *et al*., 2017; Goodall *et al*., 2013; Guan *et al*., 2016; Konishi *et al*., 2017). The results for the cisgenic lines corroborate the importance of *GS1* in maintaining grain protein concentration, as has also been highlighted through identification of QTLs in barley (Fan *et al*., 2017) and *via* a positive correlation between GS activity and grain protein content in wheat cultivars (Nigro *et al*., 2016; Zhang *et al*., 2017). Considering the fact that extra N supply cannot offset the decline in grain protein concentration of wheat plants growing in an elevated CO_2_ environment (Tausz *et al*., 2017; Walker *et al*., 2017), overexpression of *HvGS1‐1* may thus provide a means to prevent declining grain protein concentration under elevated atmospheric CO_2_.

### Concluding remarks

We conclude that the activity of cytosolic glutamine synthetase (GS1) can be increased by using a cisgenic strategy to increase expression of native *GS1* in barley. The increased GS1 activity provides an effective means of improving grain yield and NUE. Cisgenic overexpression of *GS1* may also prevent declining grain protein concentration under elevated atmospheric CO_2_.

## Experimental procedure

### Identification and isolation of HvGS1‐1

A lambda phage genomic barley library of the cultivar Igri was used for isolation of the genomic *HvGS1‐1* clone (Stratagene no. 946104; Stratagene). The library was screened using *HvGS1‐1* cDNA as probe (GenBank ID: KF815944). Subsequent isolation procedures are described in detail by Holme *et al*. (2012). The sequence used for transformation included the promoter region, the open reading frame and the terminator region (illustrated in Figure S4). The protein translated from the coding sequence completely matched with that reported by Goodall *et al*. (2013) (GenBank ID: JX878489).

### Transformation procedures

A pGreen/pSoup based vector system was used for transformation (Hellens *et al*., 2000). The vector pGreenII (http://www.pgreen.ac.uk) was engineered into a USER™ cloning vector by replacing the HpaI – StuI fragment containing the multiple cloning site between the left border and the right border with a USER cassette (Geu‐Flores *et al*., 2007). The pSoup vector providing replication functions in trans for pGreen was pClean‐S166. The pClean‐S166 vector contains a hygromycin resistance gene with a NOS promoter and a NOS terminator within its T‐DNA (Thole *et al*., 2007). The genomic *HvGS1‐1* gene was amplified by PCR from the selected lambda clone using primers with flanking USER nucleotides. The reactions were carried out using PfuTurbo^®^ CX Hotstart DNA polymerase (Stratagene) according to the manufacturer's instructions. The primer pairs amplified a 5224‐bp product of the genomic *HvGS1‐1* gene. Subsequently, the resulting PCR product was mixed with the USER™ enzyme mix (New England Biolabs) and the pre‐digested plasmid. The reaction mixture was incubated at 37 °C for 15 min followed by 15 min at 25 °C. The insertion was checked by sequencing. The *Agrobacterium tumefaciens* (updated scientific name *Rhizobium radiobacter*) AGL0 was co‐transformed with the pGreen‐HvGS1‐1 and the pClean‐S166 vectors using the freeze⁄thaw method and selected on medium with 50 mg/L kanamycin and 7.5 mg/L tetracycline according to Thole *et al*. (2007).

### Barley transformation and PCR analysis of cisgenics

The spring cultivar Golden Promise was grown in growth cabinets at 15 °C day and 10 °C night temperatures, with 16‐h light period of 400 μmol/m^2^/s. Immature embryos isolated 12–14 days after pollination were used for agrobacterium transformation following the procedure described by Holme *et al*. (2012). Regenerated plants were transferred to the greenhouse. To analyse the presence of the cisgene in the *HvGS1‐1* cisgenic plants, PCR was carried out on DNA extracted from leaf with BioSprint (QIAGEN). The oligonucleotides used in the PCR were 5′‐CGTCTGGATGCTATGGTCTC‐3′ (forward, gene specific) and 5′‐GTCAAGGGCTGAGGTTTAATAC‐3′ (reverse, gene + Pac cassette), amplifying a 238 bp fragment specific for the inserted *HvGS1‐1* copies. PCR conditions were as follow: 95 °C: 3 min; (95 °C: 30 s; 61 °C: 30 s; 72 °C; 45 s)^30 cycles^; and 72 °C: 5 min. The reaction mixture contained 1 μL of undiluted DNA extract as template, 1 unit ExTaq DNA polymerase (Clontech), 0.75 μm of each oligonucleotide, 200 μm of each dNTP, 1x ExTaq buffer and sterilized water to a total volume of 25 μL.

### Experimental plant growth conditions

T3‐generation plants of *HvGS1‐1* cisgenic lines 2.2 and 4.5 were grown together with wild‐type in six biological replicates from September 21st, 2015 until February 14th, 2016 in order to investigate the effect of N supply and elevated atmospheric CO_2_ on grain yield and N use parameters. Seeds were germinated on filter paper and subsequently transferred to 2 L pots with soil containing 0.12 g inorganic N/L soil, pH 5.6–6.4, (Pindstrup 2, Ryomgaard, DK). Three N treatments were included: low, high and excessive with 0.08 g, 0.48 g and 0.88 g inorganic N/L soil added to each pot to obtain inorganic N levels of 0.2, 0.6 and 1.0 g N/L soil, respectively. The added N was split in two equal dosages of NH_4_NO_3_ dissolved in 100 mL water and applied 28 DAG and 56 DAG. Plants were grown in two controlled greenhouses at ambient (400 μL/L) and elevated (900 μL/L) atmospheric CO_2_. Pots were randomly distributed and rotated weekly. The climate conditions in the greenhouses were set at 20 °C/16 °C day/night (16 h/8 h) temperature regime, 70% of relative humidity and of 400–500 μmol/m^2^/s photosynthetic photon flux during the day. Whole plants were harvested at maturity and separated into leaves, stem and ear, the different fractions were weighed separately and ground.

In order to provide an independent replicate of the previous experiment, T3‐generation plants of *HvGS1‐1* cisgenic lines 2.2 and 4.5 were from March 13th to July 6th 2017 grown together with wild‐type in nine biological replicates. Seed germination, soil type and pots used were the same as described above. Plants were grown in controlled greenhouse environment at ambient (400 μL/L) and elevated (800 μL/L) atmospheric CO_2_ with the same climate conditions as described above, while at a photosynthetic photon flux density of 500–600 μmol photons/m^2^/s after mid‐April. N was applied at 0.33 g N/L soil to each pot in the form of NH_4_NO_3_ solution 42 DAG, resulting in 0.45 g inorganic N/L soil. Pots were randomly distributed and were rotated every week. The two upper leaves, i.e. the flag leaf and the leaf below the flag leaf, from three main tillers of plants growing under ambient CO_2_ were collected and snap‐frozen in liquid N at 49 DAG (booting stage, before ear emergence), 70 DAG (after ear emergence) and 91 DAG (grain filling stage) for the analysis of enzyme activity. Whole plants were harvested at maturity and separated into ear and straw, weighed separately and ground.

### HvGS1‐1 gene expression analysis

Plants were grown in 1 L pots in soil with 0.12 g inorganic N available in a growth chamber at a 20 °C/16 °C day/night (16 h/8 h) temperature regime using a light intensity of 400 μmol/m^2^/s. Two youngest fully developed leaves and stems of plants were collected at 28 DAG, frozen in liquid N_2_, ground and stored at −80 °C. RNA was extracted from 100 mg of plant tissue using the TRIzol™ reagent (Thermo Fisher Scientific, catalogue no. 15596‐018). Proteins were removed by chloroform and RNA was purified by isopropanol and washed with ethanol before re‐suspension in RNase free water. Ten μg RNA was treated with DNase (New England Biolabs, catalogue no. M0303S). Synthesis of cDNA was performed on 1 μg DNase treated RNA using the M‐MuLV Reverse Transcriptase (Clontech, catalogue no. 639505). The cDNA concentration was measured on a NanoDrop spectrophotometer (Thermo Scientific, Wilmington DE) and all samples were diluted to reach the same concentration. Real‐Time qPCR was performed using 5x HOT FIREPol^®^ EvaGreen^®^ qPCR Mix Plus (ROX; Solis BioDyne, catalogue no. 08‐24‐00001) on an Mx3005P platform (Stratagene) with the following cycle: 95 °C: 3 min; (55 °C: 60 s; 59 °C: 60 s; 63 °C: 60 s)^35 cycles^, and 72 °C: 6 min. An *HvGS1.1* (GenBank ID: JX878489) specific primer pair (forward: 5′‐CCTTGTCATGTGCGATTGCT‐3′; reverse 5′‐GTACCATGGCTCCTCCTTGG‐3′) was designed to anneal across the junction of two neighbouring exons, to prevent amplification from any potential contaminating DNA. ROX was used as a passive reference dye and the comparative C_T_ (ΔΔC_T_) method was applied using the MxPro QPCR Software 4.10 (Stratagene), with expression levels normalized to actin.

### Protein extraction and GS activity assay

The frozen plant tissue was ground in liquid N_2_, and 100 mg of the ground plant tissue was extracted using 1 mL of extraction buffer containing 25 mm Tris‐HCl, 1 mm MgCl_2_, 1 mm EDTA‐Na_2_, pH 7.6; 1 mm DTT, 2% (w/v) polyvinyl pyrrolidone (PVP) and 2 mm leupeptin (Sigma Aldrich). The homogenates were centrifuged at 12 000 ***g*** for 15 min at 4 °C and the supernatant was analysed for GS activity.

Glutamine synthetase activity was assayed by the transferase reaction, which measures the ability of GS to replace the γ‐amino group of glutamine (Gln) with hydroxylamine in the presence of ADP and Na‐arsenate (Seiler *et al*., 1990). For total GS activity, 10 μL of the supernatant containing crude protein extract were incubated with 100 μL reaction buffer containing 80 mm Tris‐HCl, 64 mm Gln, 2.24 mm MnCl_2_, 25 mm Na‐arsenate dibasic, 16 mm NH_2_OH and 0.24 mm ADP, pH 6.4 for 60 min at 30 °C. Blank controls were prepared without addition of ADP and Na‐arsenate dibasic to the reaction buffer. The stop solution (0.12 m FeCl_3_, 36.4 mm TCA and 2 m HCl) was added to terminate the reaction. The product γ‐GHA (γ‐glutamyl hydroxamate) was quantified spectrophotometrically at 540 nm (FLUOstar Galaxy, BMG Labtech, Cary NC) using synthetic GHA to prepare calibration standards.

GS1 and GS2 were separated on a Mono Q 5/50 GL anion column (GE Healthcare) using Fast protein liquid chromatography (FPLC; ÄKTA, GE Healthcare, Brøndbyvester, Denmark), which was entirely kept in a cold room (4 °C). The column was pre‐equilibrated with an equilibration buffer (25 mm Tris‐HCl, 10 mm MgCl_2_, 1 mm DTT, 5% [v/v] glycerol, pH 7.0) before loading. The supernatant (250 μL) containing crude protein extract was injected onto the column and proteins were separated using two linear gradients from 0.168 to 0.246 m NaCl and 0.246 to 0.390 m NaCl at a flow rate of 0.3 mL/min. Thirty fractions of 300 μL eluate were collected after column separation and immediately assayed for GS activity. The first six eluted fractions from equilibration contained no GS proteins and were omitted from the results. The collected fractions (100 μL) were mixed with 100 μL reaction buffer and incubated at 30 °C for 60 min. Then the stop solution was added and product γ‐GHA was quantified spectrophotometrically at 540 nm (the reaction buffer, blank control and stop solution were the same as described for the total GS activity assay).

### Western‐blot analysis

Fifteen μL of each FPLC‐separated fraction was loaded on a 12% TGX Stain‐Free™ Precast gel (Bio‐Rad, Copenhagen, Denmark) and proteins were separated by gel‐electrophoresis at 180 V for 50 min in Tris/Glycine/SDS running buffer (Bio‐Rad). Separated proteins were transferred to 0.2 μm PVDF mini membranes (Bio‐Rad) using the Trans‐Blot^®^ Turbo™ Transfer System (Bio‐Rad) according to the manufacturer's instructions. Subsequently, membranes were blocked with TBS‐T (15 mm Tris‐HCl, 4.6 mm Tris base, 150 mm NaCl, and 0.1% [v/v] Tween 20, pH 7.6) containing 2.5% (w/v) skimmed milk powder. The blocked membranes were then incubated overnight with 20 mg of anti‐GS serum (rabbit IgG; Agrisera, Sweden; product no. AS08295) in 50 mL of TBS‐T at 4 °C. The antibody used recognized both cytoplasmic and chloroplastic forms of the GS enzyme (Brouwer *et al*., 2012; Silva *et al*., 2015). After several washes with TBS‐T, the membranes were incubated at room temperature for 1 h with horseradish peroxidase‐conjugated chicken anti‐rabbit IgG (1 : 5000; Agrisera, Sweden; product no. AS10833). The immune complexes were detected using a chemiluminescence reagent (Super Signal; Thermo Scientific).

### Nitrogen analysis

The N concentration in dried ground straw and grain tissue (approx. 40 mg) was analyzed by Dumas combustion (Vario Macro elemental analyzer, Elementar Analysensysteme GmbH, Hanau, Germany), using acetonitril as reference material. N harvest index (NHI) was the ratio between grain N content and total shoot N content (N content of all aboveground tissues). NUE was determined as grain dry matter produced per unit of acquired N, referred to nitrogen utilization efficiency (NUtE) by Xu *et al*. (2012). It was calculated as grain yield (g DM per plant) divided by total shoot N (g per plant). Grain protein concentration was calculated as grain N concentration multiplied by 5.4 (Mariotti *et al*., 2008).

### Statistical analysis

Statistical analysis was performed using the GLM procedure of SAS (SAS Institute 2008). For differences between genotypes in Figures 1 and 2, data were analysed by one‐way analysis of variance (ANOVA). For differences between genotypes and N levels in Figure 3 and 4, as well as differences between genotypes and CO_2_ levels in Figure 7, a two‐way ANOVA was applied. Effects of genotype, nitrogen and CO_2_ level and their interactions (Figure 6) were analysed by a three‐way ANOVA. Differences were considered statistically significant at *P* < 0.05 by Fisher's Least Significant Difference (LSD) post‐hoc test.

## EMBL accession numbers

KF815944, KF815945.

## Conflict of interest

The authors declare that they have no conflict of interest.

## Supporting information


**Table S1** Cisgene segregation pattern in *HvGS1‐1* cisgenic barley lines.
**Figure S1** Initial characterization of *HvGS1‐1* cisgenic T3 lines 2.2, 4.5, 11.1 and 11.3 together with the wild‐type.
**Figure S2** *GS1‐1* gene expression and GS1 activities in 28–day‐old plants of the two T3 *HvGS1‐1* cisgenic lines 2.2 and 4.5.
**Figure S3** Separate activities of cytosolic GS (GS1) and chloroplastic GS (GS2) in leaves of 28‐day‐old wild‐type plants.
**Figure S4** Schematic drawing of the structure of the *HvGS1.1* gene used for transformation.
