# Supplementary material for: Cisgenic overexpression of cytosolic glutamine synthetase improves nitrogen utilization efficiency in barley and prevents grain protein decline under elevated CO2
Source: Plant Biotechnol J. 2018 Dec 27;17(7):1209–21. doi: 10.1111/pbi.13046 (PMC6576097; doi:10.1111/pbi.13046)

Supplementary Table S1. Cisgene segregation pattern in *HvGS1-1* cisgenic barley lines. A) 12 cisgenic T0 lines were analysed for segregation of the inserted cisgene in the T1 generation using PCR. The resulting ratios were used to estimate the number of cisgene inserts. Cisgene detection was expected to occur at a ratio of 3:1 for a single insert line and 15:1 for a double insert line. B) T1 lines with a single cisgene insert were analysed for cisgene segregation in the T2 generation and the resulting ratios were used to determine whether the lines were homozygous or hemizygous for the inserted cisgene. Cisgene detection was expected to occur at a ratio of 1 for a homozygous line and 3:1 for a hemizygous line.

A)

Genetic background	Observed segregation				Expected no. of cisgene neg.		
T0 line	No. of T1 samples analysed	Cisgene pos.	Cisgene neg.	Ratio of cisgene pos./neg.	If single insert line	If double insert line	Evaluation
1	18	10	8	1.25:1	4-5	1-2	Single insert
2	41	29	12	2.42:1	10-11	2-3	Single insert
3	40	30	10	3:1	10	2-3	Single insert
4	41	29	12	2.42:1	10-11	2-3	Single insert
5	41	40	1	40:1	10-11	2-3	Double/multiple insert
6	41	33	8	4.13:1	10-11	2-3	Single insert
7	18	18	0		4-5	1-2	Double/multiple insert
8	18	18	0		4-5	1-2	Double/multiple insert
9	41	41	0		10-11	2-3	Double/multiple insert
10	18	18	0		4-5	1-2	Double/multiple insert
11	14	11	3	3.67:1	3-4	0-1	Single insert
12	18	18	0		4-5	1-2	Double/multiple insert

B)

Genetic background		Observed segregation			Expected no. of cisgene neg.		
T0 line	T1 line	No. of T2 samples analysed	Cisgene pos.	Cisgene neg.	If homo-zygous	If hemi-zygous	Evaluation
2	2.1	14	11	3	0	3-4	Hemizygous
2	2.2	18	18	0	0	4-5	Homozygous
3	3.2	18	18	0	0	4-5	Homozygous
3	3.4	17	13	4	0	4-5	Hemizygous
3	3.10	12	12	0	0	3	Homozygous
4	4.5	19	19	0	0	4-5	Homozygous
4	4.8	19	14	5	0	4-5	Hemizygous
11	11.1	18	18	0	0	4-5	Homozygous
11	11.2	18	15	3	0	4-5	Hemizygous
11	11.3	16	16	0	0	4	Homozygous

Supplementary Figure S1

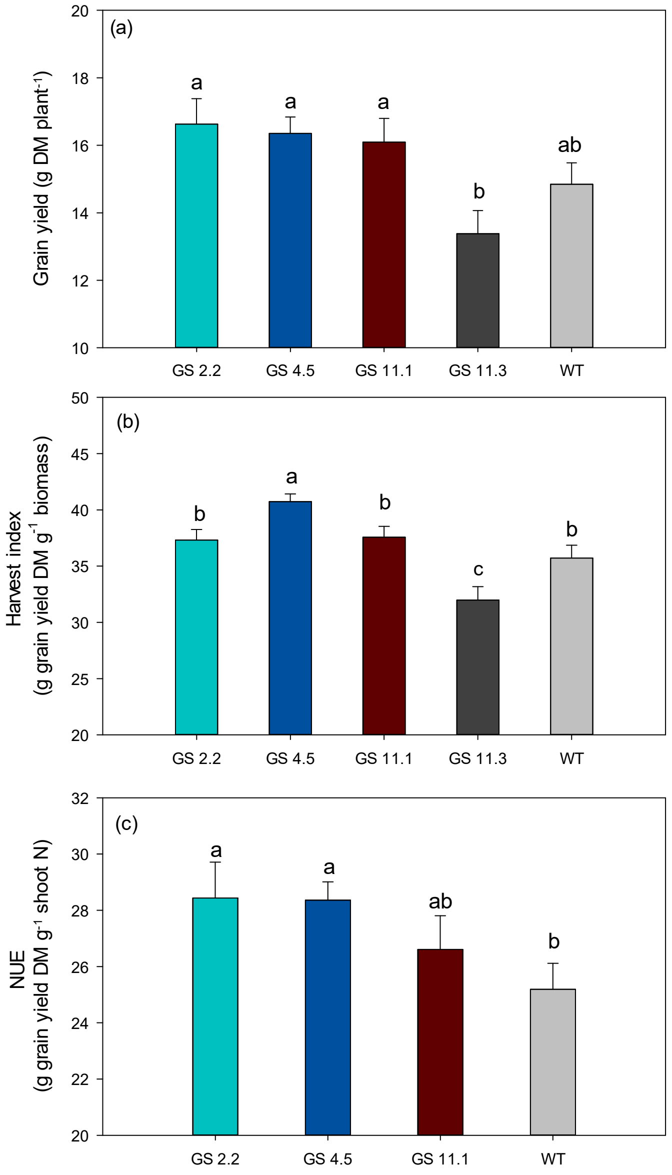

Figure S1 Initial characterization of *HvGS1-1* cisgenic T3 lines 2.2, 4.5, 11.1 and 11.3 together with the wild-type. (a) Grain yield, (b) harvest index and (c) nitrogen utilization efficiency (NUE) of plants at maturity growing with medium (0.45 g N L^{-1} soil) N supply in a greenhouse experiment conducted from December 22nd 2014 to April 10th 2015. Data are presented as means ± SE (n=20). Different letters indicate significant differences ($p<0.05$, Fischer LSD) between *HvGS1-1* cisgenic lines and wild-type.

Supplementary Figure S2

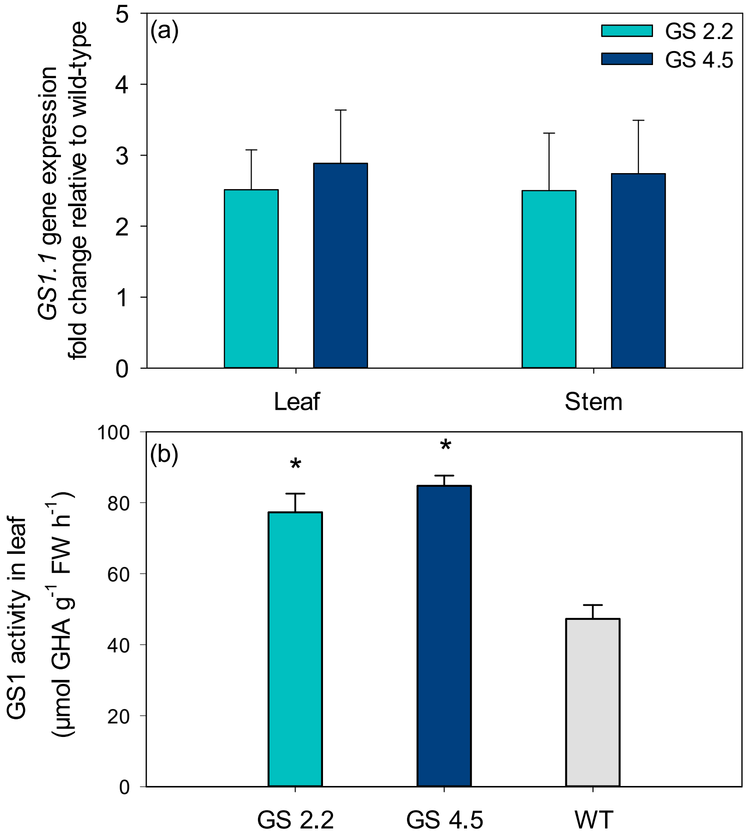

Figure S2 *GS1-1* gene expression and GS1 activity in the two youngest fully developed leaves of 28–day-old plants of the two T3 *HvGS1-1* cisgenic lines 2.2 and 4.5. (a) GS1-1 gene expression with values expressed relative to wild-type plants. (b) GS1 enzyme activity. Data represents mean values ± SE (n=4). Asterisks (*) indicates significant differences in GS1 activity between the cisgenic lines and the wild-type.

Supplementary Figure S3

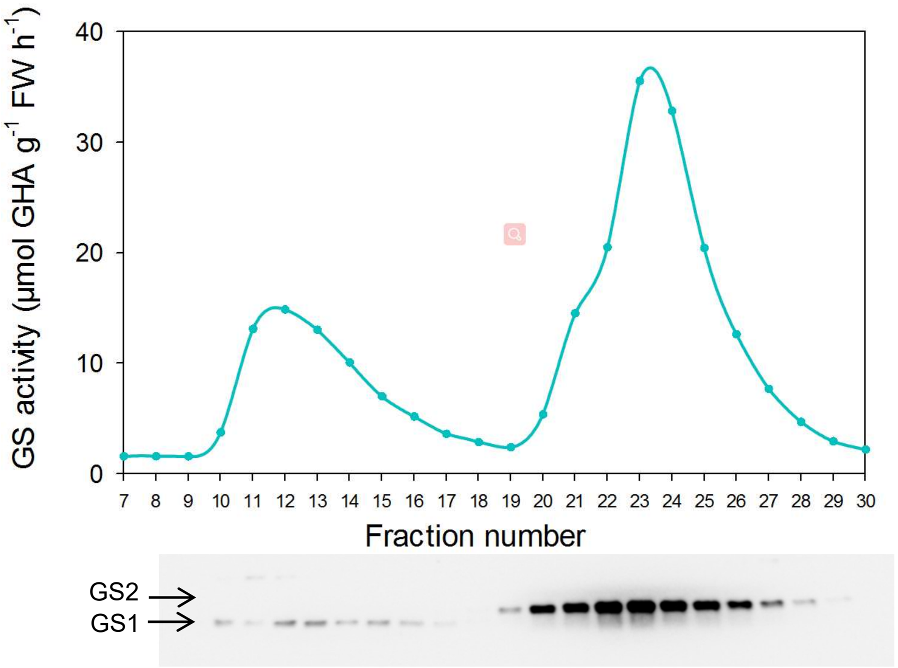

Figure S3 Separate activities of cytosolic GS (GS1) and chloroplastic GS (GS2) in leaves of 28-day-old wild-type plants. GS1 and GS2 were separated on a Mono Q 5/50 GL anion column using Fast protein liquid chromatography (FPLC). Their activities in the different elution fractions were measured by the transferase assay and the product γ-GHA (γ-glutamyl hydroxamate) was quantified spectrophotometrically at 540 nm using synthetic GHA to prepare calibration standards. The first peak corresponds to GS1 and the second peak to GS2, as confirmed by western blotting using an anti-GS antibody [GS1, lower band, 40 kDa; GS2, upper band, 45 kDa].

Supplementary Figure S4

Schematic drawing of the structure of the *HvGS1.1* gene used for transformation.

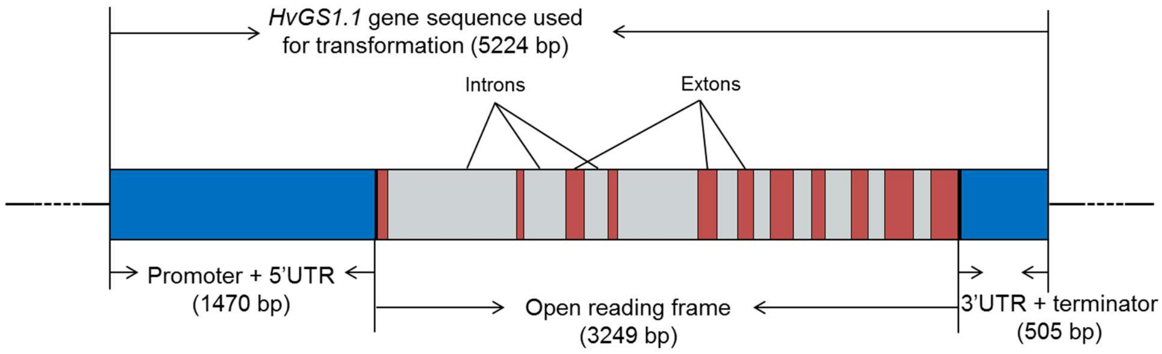

Supplement: Supplementary file 1 — Table S1 Cisgene segregation pattern in HvGS1‐1 cisgenic barley lines. Figure S1 Initial characterization of HvGS1‐1 cisgenic T3 lines 2.2, 4.5, 11.1 and 11.3 together with the wild‐type. Figure S2 GS1‐1 gene expression and GS1 activities in 28–day‐old plants of the two T3 HvGS1‐1 cisgenic lines 2.2 and 4.5. Figure S3 Separate activities of cytosolic GS (GS1) and chloroplastic GS (GS2) in leaves of 28‐day‐old wild‐type plants. Figure S4 Schematic drawing of the structure of the HvGS1.1 gene used for transformation. [file PBI-17-1209-s001.pdf]
